# Generalist practitioners’ self-rating and competence in electrocardiogram interpretation in South Africa

**DOI:** 10.4102/phcfm.v12i1.2421

**Published:** 2020-08-24

**Authors:** Langalibalele H. Mabuza, Pindile S. Mntla

**Affiliations:** 1 Department of Family Medicine and Primary Health Care, Faculty of Health Sciences, Sefako Makgatho Health Sciences University, Pretoria, South Africa; 2 Department of Cardiology, Faculty of Health Sciences, Sefako Makgatho Health Sciences University, Pretoria, South Africa

**Keywords:** generalist medical practitioners, competence, ECG interpretation, primary ECG parameters, ECG emergencies, common ECG abnormalities

## Abstract

**Background:**

Electrocardiogram (ECG) is the only practical, non-invasive method of recording and analysing cardiac abnormalities. It enables a primary healthcare (PHC) clinician to detect cardiac and non-cardiac abnormalities, some potentially life-threatening. Their early detection could save a patient’s life.

**Aim:**

The aim of this study was to evaluate the competence of generalist practitioners in ECG interpretation.

**Setting:**

This study was conducted at the Annual Refresher Course, Council for Scientific and Industrial Research (CSIR), Pretoria.

**Methods:**

A cross-sectional study was conducted amongst 93 generalist practitioners, using a self-administered questionnaire containing 20 ECG tracings, commonly encountered in PHC. The tracings were categorised into primary ECG parameters, ECG emergencies and common ECG abnormalities. Competence was determined by the generalist practitioner’s number of correctly interpreted ECG tracings. Data associations were computed using the Fisher’s exact test. Statistical significance was set at *p* ≤ 0.05.

**Results:**

Correct heart rate calculation was achieved by 14/83 (16.9%), ECG rhythm by 7/83 (8.4%), acute antero-septal myocardial infarction (MI) by 29/83 (34.9%), atrial fibrillation by 19/83 (22.9%) and cute inferior MI by 22/83 (26.5%) generalist practitioners. No correlation was found between the practitioners’ number of years in practice and competence in ECG interpretation (*p* > 0.05). The total number of correct answers achieved by all practitioners was 274/1860 (14.7%).

**Conclusion:**

The generalist practitioners had poor competency on ECG interpretation regardless of the number of years in practice. Their poor self-rating corresponded with the number of correct answers they provided. There is a need for continuous education in ECG interpretation.

## Background

Electrocardiogram (ECG) is the only practical, non-invasive method of recording and analysing cardiac abnormalities. Baseline ECG evaluation is required at primary healthcare in patients with known heart disease or dysfunction as it gives useful information regarding the presence or absence of arrhythmias, conduction defects, chamber enlargement, myocardial hypertrophy, myocardial ischemia, myocardial necrosis, pericardial inflammation and electrolytes disturbances.^1^ Electrocardiogram test may give instantaneous information that is not available with other tests.^2^ Furthermore, it is essential for patients who are to be initiated or monitored on medication with potential cardiac effects, for example, psychotropic agents (amphetamines and tricyclic antidepressants), antihypertensive drugs (beta-adrenergic receptor blockers), anti-heart failure drugs (digitalis) and others.^2^

Studies conducted in the 1990s in the United Kingdom demonstrated that doctors had a poor understanding of the ECG.^3,4^ These findings raised concern as they had a direct impact on patient safety resulting from the failure to recognise potentially life-threatening medical conditions promptly and accurately. Recommendations emanating from these study findings were made for strengthening the training of medical students on ECGs to address the identified gaps. The deficiency has persisted to the 21st century, affecting both medical practitioners and medical schools.^5,6^ In a recent study conducted amongst general practitioners (GPs) and cardiologists in the Netherlands, it was found that diagnostic accuracy of ECGs by GPs was best in atrial fibrillation, sick sinus syndrome and old myocardial infarction and poorest in incomplete right bundle branch block and left anterior fascicular block. The GPs also described false abnormalities in the ECG tracings.^7^ The implication of these study findings was that areas of poor competence amongst the GPs could be established and followed by appropriate intervention measures.

In general practice, it has been found that patients presenting with chest pain have a 5% chance of experiencing an acute coronary syndrome (ACS).^8^ It has also been found that if chest pain is caused by an ACS, urgent referral to secondary care can save the patient’s life because the condition carries a high mortality risk in the first 3 days following the event,^9^ but decreases significantly if primary percutaneous coronary intervention (PCI) is carried out promptly.^10^ Recognition of this condition by a generalist practitioner, who is usually the first contact clinician at primary healthcare, could benefit an affected patient. Furthermore, correct interpretation of the ECG in emergency situations can assist a generalist practitioner to exclude unlikely cardiac causes of chest pain, namely, gastro-oesophageal, musculoskeletal disorders or panic attacks,^2,11^ thereby allowing appropriate patient management.

At the time of this study, there was a paucity of studies conducted on the competency of medical practitioners on ECG interpretation in South Africa. The study by De Jager et al.^12^ assessed the ECG interpretation skills of South African Emergency Medicine residents at the University of Cape Town and found an overall average score of 46.4% in ECG interpretation. Larson et al.^13^ conducted a study on the feasibility of community-based ECG instruction at undergraduate MBChB programme of the University of Free State in South Africa and found that there was more support for the implementation of community-based ECG training in students’ clinical phase rather than in their preclinical phase. This finding was ascribable to the complexity of ECG interpretation requiring practical application in clinical scenarios. The current study was conducted to evaluate the competency of generalist practitioners who attended the annual refresher course organised by the Internal Medicine Department of the Sefako Makgatho Health Sciences University (SMU) in Pretoria.

## Methods

### Study design

A cross-sectional study was conducted.

### Study setting

This study was conducted on 13 September 2019 at the Council for Scientific and Industrial Research (CSIR) Conference Centre in Pretoria, Gauteng province. At the time of the study, the Department of Internal Medicine held its annual Healthcare Professionals’ Refresher Course at that venue to update medical practitioners on clinical developments in various disciplines (such as obstetrics and gynaecology, general surgery, internal medicine, family medicine, paediatrics, psychiatry and some related sub-specialties, e.g., orthopaedics). On average, the number of generalist medical practitioners in attendance was 150. There were a few additional other healthcare providers, for example, nurses who were also in attendance. These were less than five in number and thus were not the focus of this study. The medical practitioners had come from all the nine provinces of South Africa, although the majority (60% – 70%) came from Gauteng, Mpumalanga and Limpopo provinces.

### Sample size and sampling

All the generalist practitioners were requested to participate, but only 93/150 (62%) consented to participate and completed the questionnaire provided. The number of responses varied per specific question as some respondents chose not to answer certain sections of the questionnaire – hence the ‘*n*’ in the results section ranged from 40 to 93. A generalist practitioner was defined in the South African context as a medical practitioner without any further qualification in a specialised field, following obtaining his or her basic medical degree, for example, MBChB or an equivalent. In South Africa, there are various categories of generalist practitioners, namely, a medical intern (one who just qualified with the basic medical degree and is working under the supervision of senior medical practitioners), a community service doctor (1 year after competing internship), a medical officer (who completed community service and is working in the public sector) and a GP who is a medical officer practising in the private sector.^14^

### Measurement tools and data collection

Data were collected by means of a questionnaire with a sample of 20 ECG tracings obtained from the data bank of the Dr George Mukhari Academic Hospital and the main researcher’s lecture notes. These tracings were reviewed for correctness by the second author of this manuscript, who is a practising specialist cardiologist at SMU. The tracings were categorised into three groups: (1) primary ECG parameters, (2) ECG emergencies and (3) common ECG abnormalities.

In completing the questionnaire, the respondents had to interpret the primary ECG parameters (calibration, rate, rhythm and axis) and also to interpret each ECG tracing provided for emergencies and common ECG abnormalities encountered at primary healthcare. A statistician was utilised for analysis. The number of correct answers was collated per category, and the final combined score was obtained to assess the generalist practitioners’ competence in ECG interpretation. Data associations, for example, self-rating and the actual correct answers obtained, were computed by using the Fisher’s exact test. Statistical significance was set at *p* ≤ 0.05. SAS Version 9.4 (SAS Institute Inc., Carey, NC, USA) was used for the analysis.

### Ethical consideration

To adhere to the Declaration of Helsinki on ‘Ethical Principles for Medical Research Involving Human Subjects’, participation was voluntary and written informed consent was obtained from each respondent. To ensure anonymity and confidentiality, no respondent personal identifiers were used in the collected data. The data were password-protected and kept in the main researcher’s computer. Ethical clearance for the study was obtained from the Sefako Makgatho Health Sciences University Research and Ethics Committee (SMUREC) (clearance Certificate Number: SMUREC/M/222/2019:IR).

## Results

The age of the generalist practitioners ranged from 27 to 67 years, with a mean age of 49.4 years. The majority were men (65, 80%). General practitioners formed the largest proportion (58, 69.1%) of the sample. Most of the generalist practitioners (56, 72.7%) had been in practice for more than 15 years (Table 1).

**TABLE 1:** Baseline characteristics of the generalist practitioners.

Baseline characteristics	Mean	Median	s.d.	*n*	%
**1. Age (years), *n* = 40**	49.4	48	10.6	**-**	**-**
Range	-	-	-	27–67	-
Lower quartile	-	-	-	42	-
Upper quartile	-	-	-	57.5	-
**2. Sex, *n* = 81**					
Male	-	-	-	65	80.3
Female	-	-	-	16	19.8
**3. Occupation, *n* = 84**					
Private GP	-	-	-	58	69.1
Medical officer	-	-	-	10	11.9
Clinical manager	-	-	-	07	8.3
Chief executive officer	-	-	-	02	2.4
GP + MO	-	-	-	05	6.0
GP + CEO	-	-	-	01	1.2
MO + Clinical manager	-	-	-	01	1.2
**4. Years in practice, *n* = 77**					
1–5	-	-	-	01	1.3
6–10	-	-	-	05	6.5
11–15	-	-	-	15	19.5
16–20	-	-	-	25	32.5
> 20	-	-	-	31	40.2

s.d., standard deviation; GP, general practitioner; CEO, chief executive officer; MO, medical officer.

**FIGURE 1:**
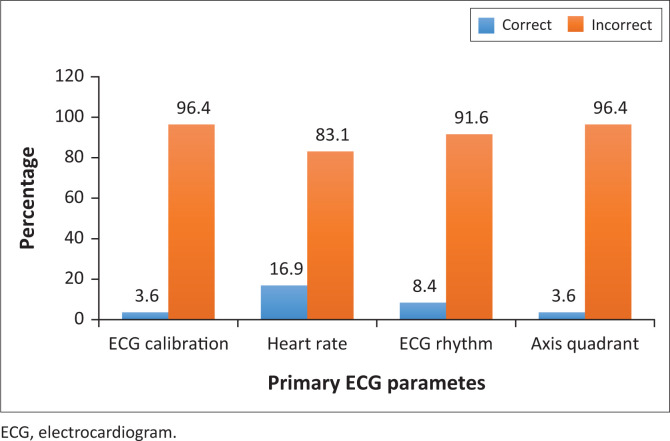
Generalist practitioners’ competence on primary electrocardiogram parameters.

Figure 1 shows that the parameter with the highest number of correct answers was the ‘heart rate’ (16.9%), whilst those with the least were the ‘ECG calibration’ and the ‘axis quadrant’ (3.6% each).

**FIGURE 2:**
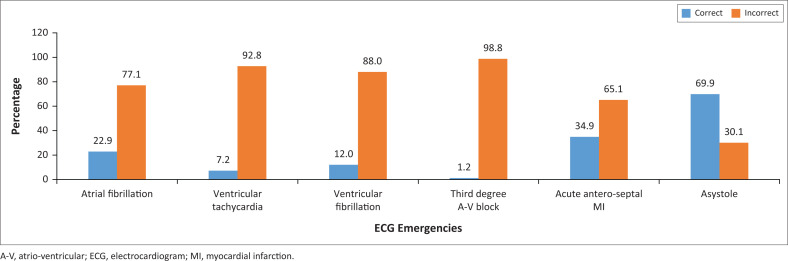
Generalist practitioners’ competence on electrocardiogram emergencies.

Figure 2 shows that the parameter with the highest number of correct answers in the ECG emergency category was the ‘asystole’ (69.9%), followed by the ‘acute antero-septal’ MI (34.9%) and lastly ‘atrial fibrillation’ (22.9%). The parameter with the least correct answers was the ‘third degree A-V block’.

**FIGURE 3:**
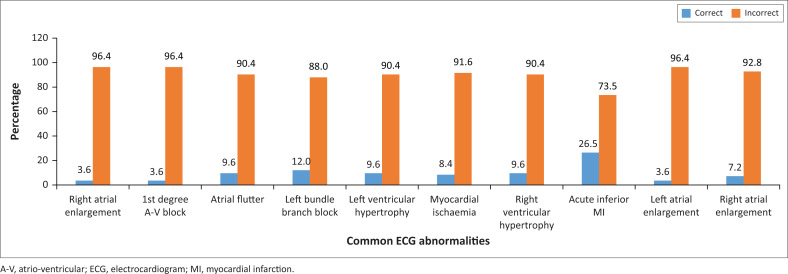
Generalist practitioners’ competence on common electrocardiogram abnormalities.

In this category, as shown in Figure 3, the parameter with the highest correct answers was the ‘acute inferior MI’ (26.5%), and the rest were about 10% or far less.

Out of the 93 respondents, only 77 (82.8%) reported their years of experience, of whom 56 (72.7%) had more than 15 years of experience. It was noted that the percentage of correct answers in the interpretation of the ECG tracings decreased in all three categories (primary ECG parameters, ECG emergencies and common ECG abnormalities) amongst the practitioners with > 15 years’ experience in practice compared to those with ≤ 15 years, although not significantly (*p* > 0.05) (Table 2).

**TABLE 2:** Proportion of correct answers versus number of years in practice (*n* = 77).

Description	Years in practice				p*
	≤ 15 21 (27.3%)		> 15 56 (72.7%)		
	*n*	%	*n*	%
**A**					0.223
Primary ECG parameters					
Total correct answers	9/84	10.7	14/224	6.2	
Total incorrect answers	75/84	89.3	210/224	93.8	
**B**					0.119
ECG emergencies					
Total correct answers	37/126	29.4	82/366	22.4	
Total incorrect answers	89/126	70.6	284/366	77.6	
**C**					0.587
Common ECG abnormalities					
Total correct answers	23/210	11	53/560	9.5	
Total incorrect answers	187/210	89	507/560	90.5	

ECG, electrocardiogram.

* , Fisher’s exact test.

Table 3 demonstrates the number of correct answers in each of the three categories (A, B and C) that were calculated as 31/372 (8.3%), 147/558 (26.3%) and 96/930 (10.3%), respectively. The total number of correct answers (combining the three categories) achieved by all the 93 participating medical practitioners was 274/1860 (14.7%). The highest number of correct answers was observed in the ECG emergencies category (147/558, 26.3%), followed by the common ECG abnormalities (96/930, 10.3%) and lastly the primary ECG parameters category (31/372, 8.3%). The category with the highest proportion of generalist practitioners who rated themselves ‘Poor’ was B (ECG emergencies; 84/342, 24.6%). The category with the highest proportion of generalist practitioners who rated themselves ‘Good’ in ECG interpretation was C (common ECG abnormalities; 6/20, 30%). Category B had the highest proportion of the generalist practitioners who did not rate themselves (24/60, 40%).

**TABLE 3:** Comparison of practitioners’ total number of correct answers per category and their self-rated competence (*n* = 93).

Description	Self-rated competence and number of correct answers given									
	Poor		Average		Good		Not rated		Total	
	*n*	%	*n*	%	*n*	%	*n*	%	*n*	%
**A**										
Primary ECG parameters	18/228	7.9	7/96	7.3	2/8	25	4/40	10	31/372	8.3
**B**										
ECG emergencies	84/342	24.6	36/144	25	3/12	25	24/60	40	147/558	26.3
**C**										
Common ECG abnormalities	34/570	6	38/240	15.8	6/20	30	18/100	18	96/930	10.3

**Total**	**136/1140**	**11.9**	**81/480**	**16.9**	**11/40**	**27.5**	**46/200**	**23**	**274/1860**	**14.7**

ECG, electrocardiogram.

Regarding the generalist practitioners who did not rate themselves, out of the 40 answers that they provided, four (10%) were correct in category A, 24/60 (40%) in category B and 18/100 (18%) in category C. The overall picture was that 46 out of 200 answers given by the practitioners who did not rate themselves were correct answers (23%).

## Discussion

This study set out to investigate the competence and self-rating on ECG interpretation of generalist practitioners who attended the annual refresher course organised by the Sefako Makgatho Health Science University in September 2019. The discussion focuses on their competence on primary ECG parameters, ECG emergencies and common ECG conditions which a generalist practitioner should be able to interpret. Furthermore, their self-rated competence has been compared with their actual performance to gain an understanding of how they also evaluated themselves.

The fact that the majority of the generalist practitioners were private GPs (about 70%) highlights the need for competence in ECG interpretation, given that they consult mainly undifferentiated patients^15^ who may present with ECG conditions warranting immediate recognition and initial treatment before referral to secondary or tertiary levels of clinical care. Medical officers, although they formed a low proportion of the practitioners (8%), constitute an important cadre group in public healthcare facilities as they also fulfil the role of first contact medical practitioners.^16^ At the time of this study, most of the generalist practitioners had been practising for more than 15 years (73%). It was noted that the advancing number of years in practice did not result in better competency in ECG interpretation; in fact, it decreased in all the three categories of the ECG interpretation which were evaluated, but not significantly. A study conducted by McCrea and Saltissi17 amongst GPs in Merseyside in the United Kingdom also found that there was no association between practice experience and proficiency in ECG interpretation, which suggested the need for ongoing refresher training in ECG interpretation for the GPs.

### Competence on primary electrocardiogram parameters

The generalist practitioners displayed a poor competence in this category. The highest number of correct answers was observed in the ‘heart rate’ interpretation, whilst the rest were poorly recognised. A medical practitioner should be able to interpret primary ECG parameters which orientate him or her as to the calibration, heart rate, axis and the rhythm. Recognising the calibration is important for the correct interpretation of the voltage in a given ECG strip. Correct interpretation of the heart rhythm could reveal clinically important heart conditions, for example, heart blocks, which cannot be identified by pulse palpation using a finger. The ECG axis can corroborate findings such as cardiac muscle enlargements and myocardial infarctions.^1^

### Competence on electrocardiogram emergencies

In the ECG emergencies category, ‘asystole’ was correctly identified by more than 50% of the generalist practitioners. Asystole is a cardiac arrest rhythm with no discernible electrical activity on the ECG monitor, thus showing a flat line ECG, where the *P* waves, QRS complexes and *T* waves are not present. It usually follows untreated ventricular tachycardia, ventricular fibrillation, bradycardia, QRS prolongation and other cardiac pathologies.^18,19^ Essentially, the heart is not functioning at that time. It is a life-threatening condition that requires immediate intervention to restart the heart function. It should be appreciated that not all generalist practitioners have 12-Lead ECG machines that may clearly delineate ventricular tachycardia or fibrillation, but all generalist practitioners can be confronted with a patient who goes into asystole during managements which needs proper recognition. There is evidence that prompt identification when it has freshly occurred is clinically important, given that patient survival following an asystole has been estimated at 5%.^18^ It was also noted that in this category, only about one-third of the generalist practitioners could recognise the antero-septal MI. The risk of death resulting from an acute MI has been found to be 12% – 15%.^20^ This implies that recognition of this condition accompanied by the necessary intervention could save about 1 in 10 patients presenting with this condition.^20^

The generalist practitioners displayed a very poor competence in recognising the other parameters in this category which were related to heart rhythms, namely, atrial and ventricular fibrillations, ventricular tachycardia and third-degree heart block. The correct identification of ECG abnormalities on rhythm, for example, atrial fibrillation, left and right bundle branch blocks and second- or third-degree A-V blocks, enables a primary healthcare practitioner to refer patients with these conditions for prompt further investigations with, for example, echocardiography in order to assess structural and functional cardiac properties.^21^ Furthermore, identification of atrial fibrillation by a generalist practitioner could enable him or her to regulate the heart rate by means of beta-blocker medication. Given the emergency nature of this category, the finding that the generalist practitioners displayed a poor competence in this category is a cause for concern and necessitates frequent continuous professional development activities to address this knowledge deficiency. The same sentiment was expressed by more than 60% of the residents in family medicine in Nigeria when their knowledge and utilisation of the ECG was found lacking.^22^

### Competence on common electrocardiogram abnormalities

The fact that the generalist practitioners could not recognise common ECG abnormalities is another cause for concern as these are the ECG tracings that should be identified at primary healthcare where generalist practitioners work.^23^ If such conditions are not identified at that level, the affected patients may miss the opportunity for referral to the secondary level of care, or even further up to tertiary or quaternary levels for further specialised management. This situation has the potential to delay patients into such extent that by the time they are discovered, complications have developed.^24^ Only about one in four generalist practitioners could recognise ‘acute inferior MI’. Namdar et al.^25^ have proven that in patients with acute inferior MI, ‘the left precordial ST-segment depression is associated with more advance coronary artery disease and worse in-hospital clinical outcomes’. A patient suffering from this condition presenting to a generalist practitioner who cannot interpret the ECG tracing has a poor prognosis, indeed.

### Generalist practitioners’ self-rated competence in electrocardiogram interpretation

There was a paucity of studies evaluating self-assessment or self-rating of medical practitioners regarding ECG competency. Literature addressing self-assessment focused on pre- and post-training evaluation of clinical skills pertaining to ECG interpretation.^26^

In this study, about two-thirds of the generalist practitioners rated themselves as having a poor ECG competency, whilst less than 3% rated themselves as having good competence. Furthermore, this study has shown that the category with the highest self-rating of ‘Poor’ was the ECG emergencies, whilst the category with the highest self-rating of ‘Good’ was the common ECG abnormalities. However, the overall picture of only about 15% correct answers provided by all the generalist practitioners paints a gleam picture of the overall competence in ECG interpretation amongst the practitioners. The category of ‘Primary ECG parameters’, which entails basic knowledge in ECG interpretation, also has a ‘Poor’ self-rating. The training in basic ECG parameters so as to properly understand an ECG tracing and contextualise the patient’s clinical condition has been stated elsewhere.^27^

Amongst the ECG tracings depicting emergencies, more than two-thirds of the generalist practitioners showed best competence in the asystole tracing, of whom about half had poorly rated their competence. This category had the highest proportion of the generalist practitioners (two out of five) who did not rate themselves. This implies that a high proportion of the generalist practitioners did not want to commit themselves regarding competence on ECG emergencies. The same poor self-rating maintained regarding the category on common ECG abnormalities.

The poor self-rating in all three categories implies that the generalist practitioners acknowledged their inefficiency in the ECG interpretation skill. Further studies are required to investigate if they had any plan to re-address the identified shortfall.

## Strength and limitations

The study methodology was quantitative, which limited further enquiry, for example, what was the basis of the poor self-rating amongst two-thirds of the respondents and good self-rating amongst the only two practitioners who rated themselves as competent. A qualitative approach would have provided in-depth understanding of this self-rating, including the explanation of what they mean by each level (‘poor’, ‘average’ and ‘good’). As far as the authors are concerned, no similar study has been conducted in South Africa on generalist practitioners’ competence level in ECG interpretation. Furthermore, although the sample size was relatively small, the fact that the respondents came from all the provinces of the country gives a fair indication of the state of affairs regarding the subject amongst the generalist practitioners in South Africa.

## Conclusion

This study has demonstrated that generalist practitioners who attended the SMU Annual Health Professionals’ Refresher Course had poor competency on ECG interpretation in the areas of primary ECG interpretation, ECG emergencies and common ECG abnormalities. There was no correlation between their competence level and the number of years they had been in practice. They rated themselves as having poor competence in ECG interpretation, which tallied well with the number of correct answers they provided. There is a need for frequent continuing professional development refresher courses on the interpretation of ECG tracings of clinical conditions commonly encountered by generalist practitioners.

## References

[1] Schlant RC, Adolph RJ, DiMarco JP, et al Committee Members Guidelines for electrocardiography. A report of the American College of Cardiology/American Heart Association task force on assessment of diagnostic and therapeutic cardiovascular procedures (Committee on Electrocardiography). Circulation. 1992;85(3):1221–1218. 10.1161/01.CIR.85.3.1221 1537123

[2] Scottish Intercollegiate Guidelines Network (SIGN) Acute coronary syndromes. A National clinical guideline. No. 93. Edinburgh: Royal College of Physicians of Edinburgh; 2007.

[3] Gillespie MD, Brett CTF, Morrison WG, Pringle SD Interpretation of the emergency electrocardiogram by junior hospital doctors. J Accid Emerg Med. 1996;13(6):395–397. 10.1136/emj.13.6.395 8947796 PMC1342806

[4] Montgomery H, Hunter S, Morris S, et al Interpretation of electrocardiograms by doctors. BMJ. 1994;309(6968):1551–1552. 10.1136/bmj.309.6968.1551 7819897 PMC2541733

[5] Matthias TA, Indrakumar J Competency of final year medical students in ECG interpretation – An experience of a medical school in South Asia. Asian Stud Med J. 2013;13(6):1–6.

[6] Jablonover S, Lundberg E, Zhang Y, Stagnaro-Green A Competency in electrocardiogram interpretation among graduating medical students. Teach Learn Med. 2014;26(3):279–284. 10.1080/10401334.2014.918882 25010240

[7] Compiet SAM, Willemsen RTA, Konings KTS, Stoffers HEJH Competence of general practitioners in requesting and interpreting ECGs – A case vignette study. Neth Heart J. 2018;26(7–8):377–384. 10.1007/s12471-018-1124-2 29882041 PMC6046661

[8] Buntinx F, Knockaert D, Bruyninckx R, et al Chest pain in general practice or in the hospital emergency department: Is it the same? Fam Pract. 2001;18(6):586–589. 10.1093/fampra/18.6.586 11739341

[9] Bruyninckx R, Van Den Bruela A, Buntinx F, et al Excess of mortality in patients with chest pain peaks in the first 3 days period after the incident and normalizes after 1 month. Fam Pract. 2010;27(6):604–608. 10.1093/fampra/cmq052 20639281 PMC2980602

[10] Baigent C, Collins R, Appleby P, et al ISIS-2: 10-year survival among patients with suspected myocardial infarction in randomized comparison of intravenous streptokinase, oral aspirin, both or neither. BMJ. 1998;316(7141):1337–1343. 10.1136/bmj.316.7141.1337 9563981 PMC28530

[11] Bruyninckx R, Aertgeerts B, Bruyninckx P, Buntinx F Signs and symptoms in diagnosing acute myocardial infarction and acute coronary syndrome: A diagnostic meta-analysis. Br J Gen Pract. 2008;58(547):105–111. 10.3399/bjgp08X277014 18307844 PMC2233977

[12] De Jager J, Wallis L, Maritz D ECG interpretation skills of South African Emergency Medicine residents. Int J Emerg Med. 2010;3(4):309–314. 10.1007/s12245-010-0227-3 21373298 PMC3047864

[13] Larson CO, Bezuidenhout J, Van Der Merwe LJ Is community-based electrocardiography education feasible in the early phase of an undergraduate medical curriculum? Health SA Gesondheid. 2017;22(1):61–69. 10.1016/j.hsag.2016.11.005

[14] HPCSA, Professional Guidelines [homepage on the Internet]. Pretoria: Health Professions Council of South Africa 2002 [cited 2019 Dec 21]. Available from: http://www0.sun.ac.za/ruralhealth/ukwandahome/rudasaresources2009/More/ProfessionalGuidelines.pdf

[15] O’Riordan M, Dahinden A, Aktürk Z, et al Dealing with uncertainty in general practice: An essential skill for the general practitioner. Qual Prim Care. 2011;19(3):175–181. 21781433

[16] Tiwari HC, Srivastav R, Khan SM Training and mentorship of medical officers to improve MCH care in public health facilities: Lessons learned from eastern Uttar Pradesh. J Family Med Prim Care. 2019;8(10):3202–3206. 10.4103/jfmpc.jfmpc_543_19 31742142 PMC6857420

[17] McCrea WA, Saltissi S Electrocardiogram interpretation in general practice: Relevance to prehospital thrombolysis. Br Heart J. 1993;70(3):219–225. 10.1136/hrt.70.3.219 8398491 PMC1025300

[18] Attin M, Feld G, Lemus H, et al Electrocardiogram characteristics prior to in-hospital cardiac arrest. J Clin Monit Comput. 2015;29(3):385–392. 10.1007/s10877-014-9616-0 25236259 PMC4420844

[19] Liang JJ, Bianco NR, Muser D, et al Outcomes after asystole events occurring during wearable defibrillator-cardioverter use. World J Cardiol. 2018;10(4):21–25. 10.4330/wjc.v10.i4.21 29707164 PMC5919889

[20] Stillman AE, Oudkerk M, Bluemke D, et al Assessment of acute myocardial infarction: Current status and recommendations from the North American Society for Cardiovascular Imaging and the European Society of Cardiac Radiology. Int J Cardiovasc Imaging. 2011;27(1):7–24. 10.1007/s10554-010-9714-0 20972835 PMC3035779

[21] Van Riet EES, Hoes AW, Limburg A, et al Strategy to recognize and initiate treatment of chronic heart failure in primary care (STRETCH): A cluster randomized trial. BMC Cardiovasc Disord. 2014;14(1):1 10.1186/1471-2261-14-1 24400643 PMC3898002

[22] Isiguzo GC, Iroezindu MO, Muoneme AS, Okeahialam BN Knowledge and utilization of electrocardiogram among resident doctors in family medicine in Nigeria. Niger J Clin Pract. 2017;20(9):1133–1138. 10.4103/njcp.njcp_374_16 29072236

[23] Santos P, Martins C, Sá L, Hespanhol A, Couto L Motives for requesting an electrocardiogram in primary health care. Ciên Saúde Colet. 2015;20(5):1549–1554. 10.1590/1413-81232015205.10062014 26017956

[24] Freeman K, Feldman JA, Mitchell P, et al Effects of presentation and electrocardiogram on time to treatment of hyperkalemia. Acad Emerg Med. 2008;15(3):239–249. 10.1111/j.1553-2712.2008.00058.x 18304054

[25] Namdar H, Imani L, Ghaffari S, et al ST-segment depression in left precordial leads in electrocardiogram of patients with acute inferior myocardial infarction undergoing primary percutaneous coronary intervention. Interv Med Appl Sci. 2018;10(4):191–197. 10.1556/1646.10.2018.19 30792911 PMC6376358

[26] Chudgar SM, Engle DL, Grochowski CO, Gagliardi JP Teaching crucial skills: An electrocardiogram teaching module for medical students. J Electrocardiol. 49(4):490–495. 10.1016/j.jelectrocard.2016.03.021 27083329

[27] Zeng R, Yue RZ, Tan CY, et al New ideas for teaching electrocardiogram interpretation and improving classroom teaching content. Adv Med Educ Pract. 2015;6(1):99–104. 10.2147/AMEP.S75316 25709515 PMC4329996

